# A Suspected Detached Pars Plana Cyst in the Vitreous Cavity

**DOI:** 10.18502/jovr.v15i4.7799

**Published:** 2020-10-25

**Authors:** Mukesh Kumar, Abhishek Varshney

**Affiliations:** ^1^Department of Glaucoma Service, C L Gupta Eye Institute, Ram Ganga Vihar, Moradabad, India; ^2^Department of Vitreoretina, C L Gupta Eye Institute, Ram Ganga Vihar, Moradabad, India

##  PRESENTATION

A 35-year-old woman presented with the chief complaint of bilateral ocular itching. There was no history of ocular trauma or previous eye surgery. Vision in both eyes was 20/20 with N6 near vision, normal anterior segment, and an intraocular pressure of 8 mmHg in both eyes. Fundus examination revealed a clear media. A 2–3 mm, oval-shaped, brown-pigmented cyst that was not mobile with ocular movement was present in the vitreous cavity of the right eye. The patient was asymptomatic at the time of examination. No treatment was performed; ultrasound biomicroscopy was advised, but the patient did not come for further evaluation.

##  DISCUSSION

Vitreous cysts can be classified as congenital or acquired. Congenital cysts are stable, oval-shaped, and not associated with any other ocular pathology. These cysts are remnants of the hyaloid vascular system.^[1]^ Non-pigmented cysts are usually located in the posterior vitreous; pigmented cysts arise from the iris or ciliary body pigment epithelium and may migrate into the anterior chamber or the vitreous cavity.^[2]^ Acquired vitreous cysts may result from ocular trauma, toxoplasmosis,^[3]^ or intermediate uveitis.^[4]^ They may be associated with high myopia and choroidal coloboma.^[5]^ Intraocular medulloepithelioma may also present as a free-floating vitreous cyst.^[6]^

Dislocated pars plana cyst in the vitreous is an uncommon finding. As this patient was asymptomatic and had no history of ocular trauma, we assumed that the cyst had been dislocated from its primary position.

**Figure 1:**
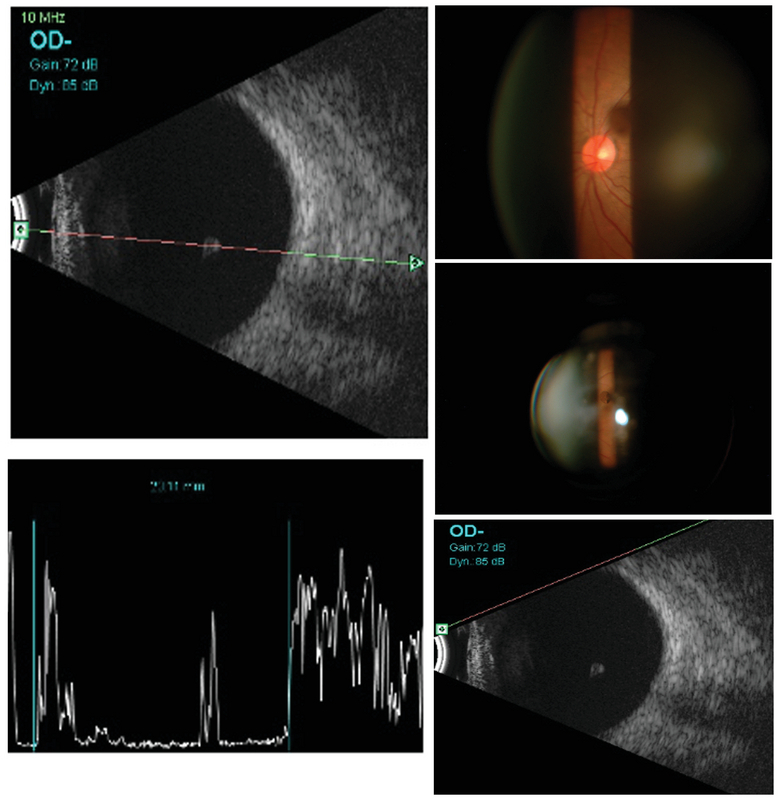
B-mode ultrasound with a 10 MHz probe showing a free-floating globule in the vitreous with a hyperechoic surface, and hypoechoic interna, suggestive of a vitreous cyst. Corresponding A-mode ultrasound shows to be normal. Slit lamp biomicroscopy photographs showing a cyst in the vitreous and a normal posterior pole of the right eye.

##  Financial Support and Sponsorship

Nil.

##  Conflicts of Interest

There are no conflicts of interest.
